# Nest attendance, incubation constancy, and onset of incubation in dabbling ducks

**DOI:** 10.1371/journal.pone.0286151

**Published:** 2023-05-19

**Authors:** C. Alex Hartman, Joshua T. Ackerman, Sarah H. Peterson, Brady Fettig, Mike Casazza, Mark P. Herzog

**Affiliations:** U.S. Geological Survey, Western Ecological Research Center, Dixon Field Station, Dixon, California, United States of America; University of Veterinary Medicine Vienna: Veterinarmedizinische Universitat Wien, AUSTRIA

## Abstract

In birds, parents must provide their eggs with a safe thermal environment suitable for embryonic development. Species with uniparental incubation must balance time spent incubating eggs with time spent away from the nest to satisfy self-maintenance needs. Patterns of nest attendance, therefore, influence embryonic development and the time it takes for eggs to hatch. We studied nest attendance (time on the nest), incubation constancy (time nests were at incubation temperatures), and variation in nest temperature of 1,414 dabbling duck nests of three species in northern California. Daily nest attendance increased from only 1–3% on the day the first egg was laid to 51–57% on the day of clutch completion, and 80–83% after clutch completion through hatch. Variation in nest temperature also decreased gradually during egg-laying, and then dropped sharply (33–38%) between the day of and the day after clutch completion because increased nest attendance, particularly at night, resulted in more consistent nest temperatures. During the egg-laying stage, nocturnal nest attendance was low (13–25%), whereas after clutch completion, nest attendance was greater at night (≥87%) than during the day (70–77%) because most incubation recesses occurred during the day. Moreover, during egg-laying, nest attendance and incubation constancy increased more slowly among nests with larger final clutch sizes, suggesting that the number of eggs remaining to be laid is a major driver of incubation effort during egg-laying. Although overall nest attendance after clutch completion was similar among species, the average length of individual incubation bouts was greatest among gadwall (*Mareca strepera*; 779 minutes), followed by mallard (*Anas platyrhynchos*; 636 minutes) and then cinnamon teal (*Spatula cyanoptera*; 347 minutes). These results demonstrate that dabbling ducks moderate their incubation behavior according to nest stage, nest age, time of day, and clutch size and this moderation likely has important implications for egg development and overall nest success.

## Introduction

Avian nest success is dependent on parents providing a safe thermal environment for their eggs that is suitable for embryonic development [1–3]. Most birds achieve this through active brood-patch contact incubation, in which heat is transferred from the incubating adult to eggs within the nest [4]. Consequently, patterns of parental nest attendance and incubation constancy can influence the rate of embryonic development. Prolonged parental absence can cause egg temperatures to decrease below what is suitable for embryonic development, thereby lengthening the incubation period and increasing exposure of eggs to predation, and also potentially reducing egg hatching success and hatchling fitness [5–10]. Greater incubation constancy would provide a more consistent thermal environment for eggs, allowing for embryos to develop more quickly, resulting in a shorter nesting period, less time spent in the vulnerable egg stage, and lower risk of predation to the eggs and the incubating adult [10,11]. For species in which the male and female take turns incubating eggs, near constant incubation may be possible [12,13], thereby minimizing the time it takes for eggs to develop and hatch. In contrast, for species in which only one parent incubates, constant incubation often is not possible and individuals must balance incubation time necessary to provide a suitable thermal environment for their eggs with periodic incubation recesses to forage and maintain their own physical condition [1,2,14,15].

In dabbling ducks, incubation of the eggs is done solely by the female [16] and hens typically take one or more incubation recesses per day, during which they leave eggs unattended for up to several hours [16–18]. For example, after clutch completion, mallard (*Anas platyrhynchos*) and gadwall (*Mareca strepera*) average 1.7 and 1.4 incubation recesses per day, respectively, and these recesses last on average, 91–193 minutes depending on the species and the time of day [18]. Previous research has demonstrated that dabbling duck hen nest attendance and incubation rhythms can be influenced by a wide variety of factors including ambient temperature, time of day, date, nest age, food availability and nutrient limitation, hen body condition, predation risk, disturbance, and weather [16,18–23]. Thus, incubating hens appear to be responsive to changing physiological and environmental conditions and moderate their incubation behavior accordingly.

The optimal amount of time hens spend incubating eggs can vary between the egg-laying stage and the incubation stage after clutch completion. During the incubation stage, greater incubation constancy is advantageous, as it may shorten the time necessary for eggs to fully develop and therefore minimize exposure of eggs and the hen to predation [10,11]. Further, because all the eggs are present after clutch completion, incubation time during this stage applies equally to all the eggs within the clutch, although within-clutch variance in egg temperature can still occur due to the physical position of eggs in the nest [24]. In contrast, incubation during the egg-laying stage could be detrimental, potentially resulting in unequal embryonic development rates among eggs in the clutch and thus hatching asynchrony, because eggs laid early in the laying sequence would receive more incubation time than eggs laid late in the laying sequence. For precocial species like dabbling ducks, in which the brood typically leaves the nest together within 24 hrs after hatch [25,26], a high degree of hatching asynchrony could delay brood departure from the nest, potentially increasing predation risk to young ducklings, or result in the abandonment of ducklings from late-hatched eggs, thereby reducing reproductive success. Although some incubation during egg-laying may be necessary to maintain egg viability [27], the onset of incubation and the degree to which incubation time varies among eggs in a clutch may influence development, hatching synchrony, and overall reproductive success.

Dabbling ducks typically lay large clutch sizes of 8–14 eggs, at a rate of one egg laid per day [28]. Thus, the 1^st^ egg in a 10-egg clutch is laid 9 days before the 10^th^ egg is laid. Yet, eggs within a clutch typically hatch within 24 hours of one another [16,25,26]. Previously it was assumed that hatching synchrony was achieved by the hen delaying incubation until clutch completion. However, more recent studies have documented that eggs reach temperatures suitable for embryonic development as early as when the second egg is laid [29], suggesting that other mechanisms exist for limiting differential development among eggs associated with their position in the laying sequence. Previous studies have found evidence of both physiological and behavioral mechanisms for maintaining developmental and hatching synchrony despite unequal incubation time among eggs due to their position in the laying sequence. In species with declining egg size with position in the laying sequence, it has been posited that smaller eggs require less developmental time than larger eggs and this helps maintain hatching synchrony [30,31]. In mallards, metabolic rate was greater for eggs laid late in the laying sequence compared to those laid early in the laying sequence, despite no relationship between egg size and position in the laying sequence [32]. Wood duck (*Aix sponsa*) eggs maintained at intermediate temperatures in incubators showed that eggs laid late in the laying sequence developed faster and required a shorter incubation period than early laid eggs [33]. Additionally, vocalizations by early laid, and thus more developed embryos, stimulating accelerated development of later laid and less developed sibling embryos has been suggested as a means for promoting hatch synchrony [34]. Finally, several studies have found variation in temperature among eggs in the clutch according to their physical position within the nest [24,35], and that later laid eggs may be more centrally located than earlier laid eggs [36] promoting faster development.

We evaluated patterns of nest attendance, incubation constancy, incubation bout duration, and nest temperature variation among three species of dabbling ducks nesting in northern California. Following previous work, we predicted that both daily hen nest attendance and incubation constancy would be low at the time of nest initiation, increase during the egg-laying stage, and then level off at the highest values during late incubation and continuing through hatch. Coinciding with these predicted increases in nest attendance and incubation constancy, we expected that variation in nest temperature would decrease as egg-laying progressed and would be considerably lower after clutch completion as hens maximize time spent incubating. Finally, we examined the potential for hens to modify incubation constancy according to the final clutch size. We predicted that hens with larger final clutch sizes would reduce nest attendance and incubation constancy during egg-laying relative to hens with smaller final clutch sizes, but then would increase nest attendance and incubation constancy after clutch completion.

## Materials and methods

### Study area and species

We studied dabbling duck incubation on and adjacent to the Grizzly Island Wildlife Area in Suisun Marsh, California (38.141°N, 121.970°W) during 2015–2019. The Grizzly Island Wildlife Area is a 3,600-ha area operated by the California Department of Fish and Wildlife, and includes 1,600 ha of managed uplands and seasonal wetlands that support hundreds of dabbling duck nests annually [37–39]. We focused on the three most common nesting dabbling duck species: mallard, gadwall, and cinnamon teal (*Spatula cyanoptera*). During 2015–2019, mallard (58%) and gadwall (39%) comprised the vast majority of duck nests in the area, whereas cinnamon teal accounted for only about 3% of nests. All three species nested concurrently, although mallard began nesting earliest (median nest initiation date: 26 April), followed by cinnamon teal (30 April), and then gadwall (9 May).

### Nest searching and monitoring

To locate duck nests, we used nest searching techniques modified from McLandress et al. [38], which consisted of dragging a 50 m rope and cans between 2 slow-moving all-terrain vehicles across upland fields. When the passing rope caused a hen to flush, we immediately stopped and searched the area to locate the nest. We determined the duck species by visual identification of the flushing hen as well as the size and color of the eggs. We marked nests with a 2 m-tall bamboo stake placed ~4 m north of the nest bowl, and a second bamboo stake no taller than the surrounding vegetation placed just outside the south edge of the nest bowl. We revisited nests approximately every 7 days until they hatched or failed. During each visit, we candled all eggs within the nest to determine the stage of embryo development (accuracy approximately ± 1 day) and the age of the nest [40] and to estimate the clutch completion date (the date the last egg was laid). For nests found during egg-laying, we estimated the clutch completion date by counting forward, assuming one egg was laid per day, until the final clutch size was reached (final clutch size was determined from subsequent visits to the nest). For nests found after clutch completion, we subtracted the average incubation stage, determined by candling, of all eggs in the nest from the date of nest discovery. We then calculated the nest initiation date (the date the first egg was laid) for each nest by subtracting the final clutch size from the estimated clutch completion date. At the end of each nest visit, we covered the eggs with down feathers and other nesting materials as hens typically do when taking an incubation recess.

### Measuring nest attendance and temperature

We used Thermochron iButton temperature dataloggers (Model DS1922L-F5#, Maxim Integrated Products, Sunnyvale, CA, USA) to record nest temperature at dabbling duck nests. Prior to deployment, we programmed iButtons to record temperature at 4-minute (2015) or 8-minute intervals (2016–2019). The 8-minute interval used in 2016–2019 allowed for one iButton to record temperature over the life of almost all nests from egg-laying through hatch. The 4-minute interval used in 2015 required the iButton be replaced at least once to prevent the memory from becoming full. We subset the 2015 data to 8-minute intervals for direct comparability with 2016–2019 data. We placed one iButton in the center of the nest bowl to measure nest temperature and placed a second iButton immediately south of the nest bowl to record local ambient temperature. Each iButton was secured within, and protruding slightly above, a cream-colored rubber stopper, attached to a metal stake that was used to anchor the unit into the ground. This arrangement allowed the iButton to remain fixed in the center of the nest at the same height as the apical surface of the eggs.

We identified hen presence and absence from the nest using an automated recess detection method as described by Croston et al. [41]. This method uses monotonic decreases in nest temperature relative to each nest’s daily variation in temperature to identify incubation recesses (hen absence from the nest) and subsequent nest temperature increases following these events signify the hen’s return at the end of an incubation recess (hen presence). The automated recess detection method works by identifying periodic temperature decreases among high and fairly constant nest temperatures and assigning them as incubation recesses. As such, it is accurate at assigning hen presence and absence after clutch completion (97% accuracy rate confirmed using videography, [41]), when hen attendance at the nest is high, but is less accurate at assigning hen presence and absence during egg-laying, when hen attendance at the nest can be low and sporadic. To address this, we visually examined daily nest temperature plots of all nests during egg-laying to proof and correct assignments of hen presence and absence. Using this approach, we identified the proportion of time a hen was on the nest each day (nest attendance), the timing and duration of each incubation bout (continuous stretch of time a hen was on the nest), and the timing and duration of each incubation recess during the egg-laying stage and the incubation stage between clutch completion and hatch. From these data we further calculated 1) the coefficient of variation (CV; standard deviation/mean) in nest temperature for each nest on each day and 2) the variation in nest temperature for each nest on each day during the time the hen was attending the nest.

In birds, egg temperatures of ~35–40°C are considered optimal for embryonic development [3], and even small decreases below this optimum can slow embryo development, decrease embryo survival, reduce chick condition at hatch, and reduce chick growth rates [3,7,8,42]. We were interested in differentiating hen nest attendance when eggs were heated to temperatures likely to best support embryo development (hereafter incubation constancy) from hen nest attendance when eggs were not heated to such temperatures. Although iButton temperature dataloggers were effective at determining nest attendance through relative changes in nest temperature over time, they did not adequately measure internal egg temperatures. In fact, average nest temperatures recorded when the hen was present at the nest after clutch completion were 22–39°C (50% central span: 32–36°C) among individual nests. This variation in temperature was more likely due to differences in hen incubation posture and/or incomplete contact between the iButton and the hen’s brood patch, than actual differences in average egg temperatures among nests during active incubation. As a result, it was not possible to use the proportion of time nests reached a fixed temperature value to measure incubation constancy. Instead, we examined the proportion of time each nest was at or above nest-specific temperatures observed during the incubation stage. We calculated the overall standard deviation for nest temperature when hens were present among all nests by species (mallard SD: 3.1°C, gadwall SD: 3.0°C, cinnamon teal SD: 3.9°C) and then subtracted this species-specific overall standard deviation from each individual nest’s specific average nest temperature when the hen was present to obtain a lower bound for the typical incubation temperature for each nest. To calculate the overall standard deviation for nest temperatures by species as well as nest-specific average nest temperatures during incubation, we used only temperature data collected when the hen was present and after clutch completion. In this way we could determine how often during the egg-laying stage each hen heated her eggs to the nest-specific temperature observed during the incubation stage. We excluded data within one day of the hatch date, because hen incubation behavior, and thus nest temperature, changed once eggs begin hatching. In addition, because it sometimes took time for eggs and the iButton to heat up once hens returned from an incubation recess, we also excluded the first 2 nest temperature data points, representing the first 16 minutes of an incubation bout, so that lower temperatures during these heating-up periods were not included in calculations of the nest temperature means and overall standard deviation when hens were present. By examining the proportion of time nests were at or above these nest-specific temperatures, we were able to examine how often eggs were exposed to incubation temperatures during the egg-laying while accounting for differences in recorded temperatures among individual nests due to iButton placement and contact with the hen. The underlying assumption is that average temperatures recorded at each nest during the time the hen was present and after clutch completion represented nest-specific temperatures typical for egg development.

### Variation in nest attendance and incubation constancy by time of day

We further divided each day into daytime, nighttime, and nautical twilight components using solar zenith angles (daytime: <90°, nighttime: >102°, and nautical twilight: ≥90° and ≤102°). Solar zenith angles for each minute of each day of the study period were obtained from the NOAA Antarctic UV Monitoring Network Solar Geometry Calculator (https://www.esrl.noaa.gov/gmd/grad/antuv/SolarCalc.jsp) using the latitude and longitude of the approximate center of the study area (38.141° N, 121.970° W). In addition, we expanded nest stage into 3three levels: egg-laying, early incubation (1–13 days after clutch completion), and late incubation (14–26 days after clutch completion).

### Statistical analyses

We investigated daily nest attendance (percent time on the nest), incubation constancy (percent time nests were at nest-specific incubation temperatures), and variation in nest temperature of dabbling duck nests during the egg-laying stage and the incubation stage after clutch completion. To prevent continuous nighttime hours (~20:00–04:00 hrs) from being split into 2 separate 24-hr periods, we defined a day as beginning at 04:00 and ending at 04:00 on the following day. For each nest, we determined the last day the nest was attended by the hen by visual assessment of nest temperature plots and excluded that day and all subsequent days from analyses. We removed any nest-days beyond 26 days after clutch completion (as most nests hatch at 25–26 days after clutch completion) and we removed the day before and the day of hatch for nests that hatched, because incubation behavior changed around the time of hatch. In addition, for each nest we excluded all data from days that a nest was visited by investigators and data on and after the date a nest or hen was manipulated as part of companion studies (such as attaching transmitters to hens; [43,44]), which only occurred during the incubation stage after clutch completion. We also excluded nests that contained only dead or infertile eggs, and nests that did not survive to clutch completion (because final clutch size could not be determined). Finally, only nest-days that included the full 24 hours of temperature data (04:00–04:00) were included.

#### Mallard and gadwall

We used separate linear mixed models (PROC MIXED, SAS/STAT software release 9.4, SAS Institute, Cary, North Carolina, U.S.A.) by species to evaluate the effects of several variables on 1) daily nest attendance, 2) daily incubation constancy, 3) daily variation in nest temperature (full 24 hours), and 4) daily variation in nest temperature when the hen was attending the nest. We used a logit transformation of the response variable for 1) daily nest attendance and 2) daily incubation constancy, and we used a natural log transformation of the response variable for 3) daily variation in nest temperature and 4) daily variation in nest temperature when the hen was attending the nest, as the residuals from models without these transformations were not normally distributed. Half the minimum non-zero value was added when the response variable was zero before the logit or log transformations, and half the minimum non-zero value was subtracted when the response variable was one (logit transformations only). Explanatory variables included the categorical variable nest stage (egg-laying stage vs. incubation stage), and continuous covariates nest initiation date (as day of year where January 1 = Day 1), the final clutch size of the nest once egg-laying was complete (representing the total number of eggs laid by the hen and did not account for any subsequent egg loss due to partial depredation of the clutch), and nest age (where nest age = 1 on the day the first egg was laid). We modeled nest age as either a linear, quadratic, or cubic trend, and also evaluated a nest stage×nest age interaction in models with a linear trend for nest age to allow slopes for nest age to vary between the egg-laying stage and the incubation stage. We did not include a nest stage×nest age interaction term when evaluating quadratic or cubic trends for nest age because the quadratic and cubic trends already accounted for potential changes in slope between the egg-laying and incubation stages. For each species-specific analysis, we built a balanced set of all combinations of the categorical variable nest stage; a linear trend for final clutch size; linear and quadratic trends for nest initiation date; linear, quadratic, and cubic trends for nest age; the interactions nest stage×final clutch size, final clutch size×nest initiation date, and nest stage×nest age (models with linear trend for nest age only); and a null model (intercept and random effects only, no fixed effects) for a total of 97 models. In all models, we included the individual nest as a random effect and an autoregressive covariance structure to account for nonindependence of equally spaced daily repeated measures at the same nest.

Next, we conducted separate linear mixed models (PROC MIXED, SAS/STAT) by species to examine the duration of individual incubation bouts (continuous stretch of time a hen was on the nest). For this analysis, we excluded all incubation bouts for which the duration was longer than a full day (1,440 minutes; 2% of all incubation bouts). Whereas some incubation bouts may have legitimately exceeded these limits, others exceeded these limits only because a recess was not detected (missed recess detection), resulting in an erroneously long incubation bout. The response variable was incubation bout duration, modeled as the percent time of the day that the bout lasted. We used a logit transformation of the response variable as residuals from models without this transformation were not normally distributed. For each species, we built a balanced set of all combinations of the categorical variable nest stage (egg-laying stage vs. incubation stage); a linear trend for final clutch size; linear and quadratic trends for nest initiation date and ambient temperature at the beginning of the incubation bout (ambient temp); linear, quadratic, and cubic trends for nest age; the hour the incubation bout began; and the interactions nest stage×final clutch size, final clutch size×nest initiation date, and nest stage×nest age (models with linear trend for nest age only); and a null model (intercept and random effects only, no fixed effects) for a total of 330 models. We converted the hour the incubation bout began from a circular to a linear variable by dividing by 24 and multiplying by 2π, and then calculating the sine (sinHour) and cosine (cosHour) of these values. In all models, we included the individual nest as a random effect to account for nonindependence of repeated measures at the same nest.

#### Cinnamon teal

Due to a smaller sample size, we limited our analyses for cinnamon teal to 1) daily nest attendance, 2) daily incubation constancy, and 3) incubation bout duration, and built simpler models that only included all combinations of the variables nest stage, linear, quadratic, and cubic trends for nest age, and the interaction nest stage×nest age (models with linear trend for nest age only) and a null model (intercept and random effects only, no fixed effects) for a total of 9 models.

For all analyses, we ranked models according to maximum parsimony using an information-theoretic approach [45] and second-order Akaike’s Information Criterion (AIC_c_) in which the difference in AIC_c_ values (ΔAIC_c_) between the best model (lowest AIC_c_) and each other model was used to assign model rank. We used Akaike model weights (*w*) to represent the relative likelihood of each model given all the models in the candidate set. To account for model selection uncertainty, we calculated predicted values for each model using the model-specific coefficients, weighted these predicted values by the model weight, and then averaged these weighted predicted values to obtain model-averaged predictions. Unless otherwise indicated, model predictions were made at the median value of all non-focal parameters (final clutch size: mallard: 9, gadwall: 10, cinnamon teal: 10; nest initiation date as day of year: mallard: 115, gadwall: 129).

### Ethics statement

This study was approved by the U.S. Geological Survey Western Ecological Research Center Animal Care and Use Committee (written consent).

## Results

During 2015–2019, we collected nest temperature data from 1,414 nests (726 mallard, 643 gadwall, and 45 cinnamon teal) over 12,787 nest-days (6,798 mallard, 5,466 gadwall, and 523 cinnamon teal; mean±SD: 9.0±5.5 days per nest, range:1–28 days per nest) after excluding nests and nest-days for reasons described above. This included 1,005 nest-days at 356 nests (205 mallard, 135 gadwall, and 16 cinnamon teal) during the egg-laying stage and 11,782 nest-days at 1,411 nests (724 mallard, 642 gadwall, and 45 cinnamon teal) during the incubation stage after clutch completion. Mean final clutch size was 9.6 eggs (range: 5–14 eggs) for mallard, 10.6 eggs (range: 5–15 eggs) for gadwall, and 11.1 eggs (range: 8–14 eggs) for cinnamon teal.

### Daily nest attendance and incubation constancy

**Fig 1:**
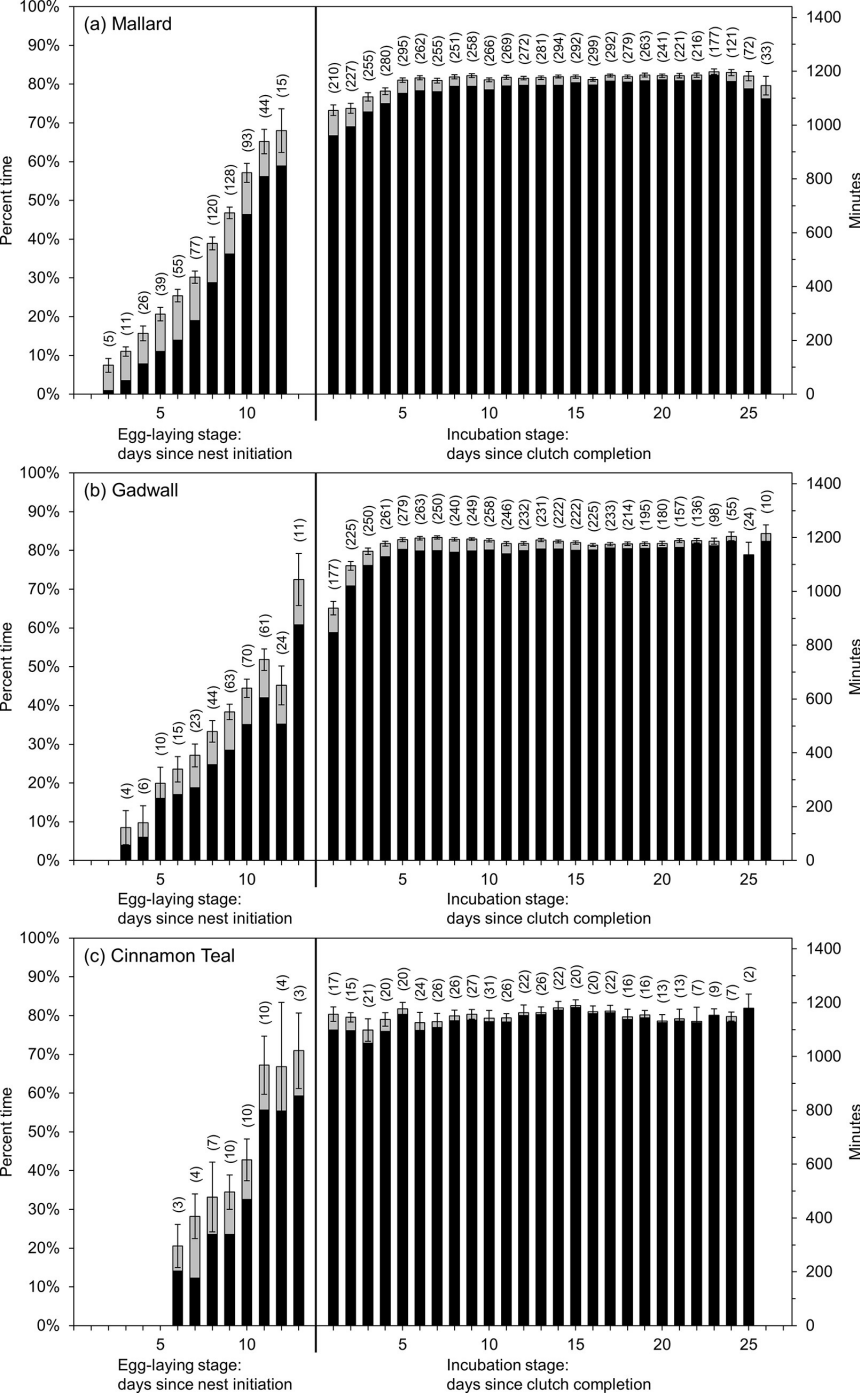
Raw data means ± standard error for the percent time (left y-axis) and corresponding minutes in the day (right y-axis) of nest attendance (black + gray bars) and incubation constancy (black bars only) for (a) mallard, (b) gadwall, and (c) cinnamon teal nests by nest age during the egg-laying stage and incubation stage in Suisun Marsh, California, 2015–2019. Sample sizes for each nest age are in parentheses. Nest age = 1 during the egg-laying stage denotes the day the first egg was laid. Nest age = 1 during the incubation stage denotes the first day after clutch completion.

**Fig 2:**
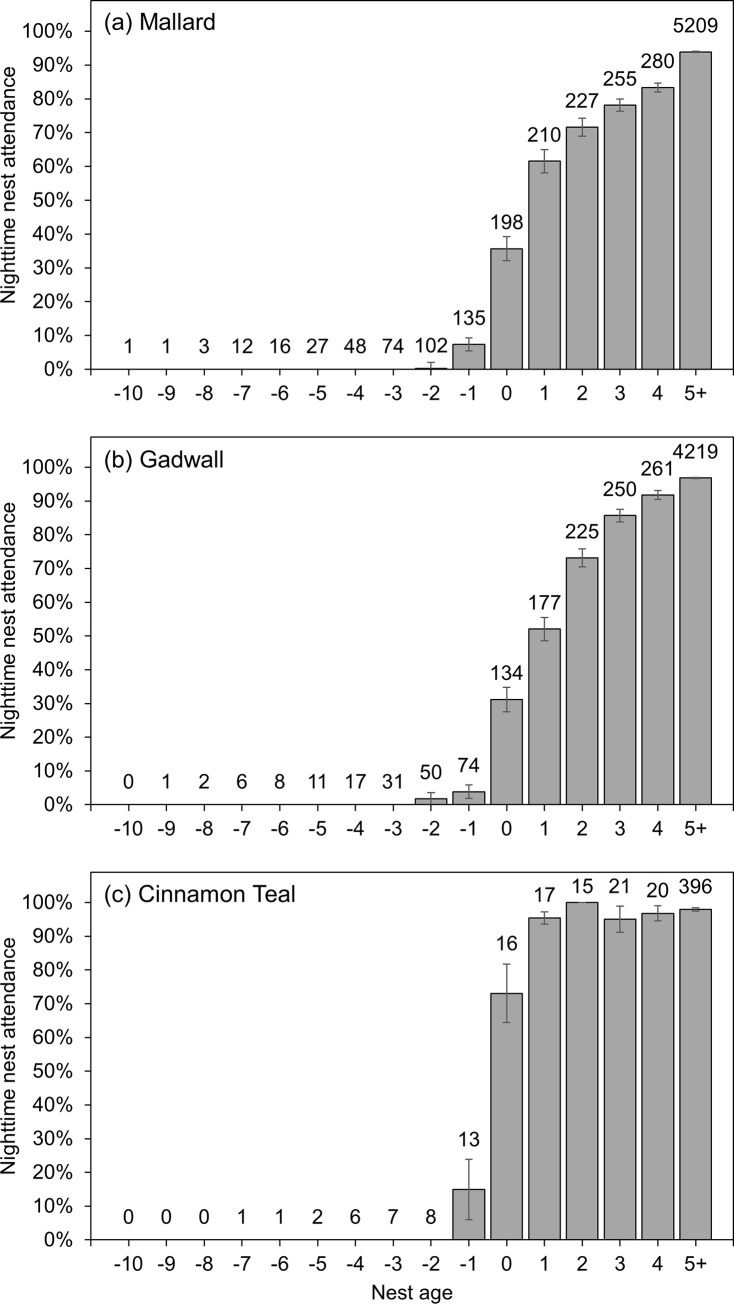
Raw data means ± standard error nighttime nest attendance (solar zenith angle>102°) for (a) mallard, (b) gadwall, and (c) cinnamon teal in Suisun Marsh, California, 2015–2019. Nest age = 0 corresponds to the estimated clutch completion date, negative values for nest age represent days prior to clutch completion, and positive values represent days after clutch completion. Sample sizes for each nest age are provided above the bars.

Nest attendance and incubation constancy increased gradually with nest age throughout the egg-laying stage in all three species, followed by a large increase after clutch completion before levelling off at daily rates at or above 80% for both measures until hatch (Fig 1). The gradual increase during the egg-laying stage was the result of hens increasing diurnal nest attendance as egg-laying progressed and the large increase after clutch completion coincided with the onset of overnight incubation. In all three species, hens spent no time (0%) incubating at night throughout most of the egg-laying stage but increased nocturnal incubation time to over 50% (mallard), 60% (gadwall), and 95% (cinnamon teal) immediately after clutch completion (Fig 2).

For mallard and gadwall, the variables nest stage, nest age, final clutch size, and nest initiation date were all important predictors of nest attendance (S1 Table). For mallard, models that included a linear trend for nest age and a nest stage×nest age interaction performed better than models that included a cubic term for nest age. In contrast, for gadwall, models with a cubic trend for nest age performed better than models with a linear trend for nest age and a nest age×nest stage interaction S1 Table). For mallard, only the top-ranked model was competitive (ΔAIC_c_≤2.0) and included nest stage, linear trends for nest age and final clutch size, a quadratic trend for nest initiation date, and the interactions nest stage×nest age, nest stage×final clutch size, and final clutch size×nest initiation date. For gadwall, the top-ranked model included nest stage, a linear trend for final clutch size, a quadratic trend for nest initiation date, a cubic trend for nest age, and a nest stage×final clutch size interaction. A second gadwall model also was competitive (ΔAIC_c_ = 1.85) and was the same as the top-ranked model but also included the interaction final clutch size×nest initiation date. For cinnamon teal, the top-ranked model included nest stage, a linear trend for nest age, and the interaction nest stage×nest age (S1 Table); no other models were competitive.

Model selection results for incubation constancy were similar to the results for nest attendance (S2 Table). The top-ranked models for mallard and cinnamon teal incubation constancy were equivalent to the respective top-ranked models for nest attendance, and no other models were competitive (ΔAIC_c_≤2.0). The top-ranked model for gadwall incubation constancy was equivalent to the top-ranked model for gadwall nest attendance, and only the second-ranked model for gadwall incubation constancy, which was equivalent to the second-ranked model for gadwall nest attendance, was competitive (ΔAIC_c_ = 1.75).

#### Egg-laying stage

Mean (95% confidence interval) model-predicted values showed that daily nest attendance for mallard nests increased from 3% (2%, 3%) on the day the first egg was laid (nest age = 1) to 55% (52%, 57%) on the estimated day of clutch completion (nest age = 9). Daily nest attendance for gadwall nests increased from 1% (0.5%, 1.4%) on the day the first egg was laid (nest age = 1) to 51% (48%, 54%) on the day of clutch completion (nest age = 10). For cinnamon teal, daily nest attendance increased from 1% (0.7%, 3%) on the day the first egg was laid (nest age = 1) to 57% (51%, 62%) on the day of clutch completion (nest age = 10). Similarly, incubation constancy increased as egg-laying progressed, from 0.3% (0.2%, 0.4%) on the day the first egg was laid (nest age = 1) to 45% (42%, 48%) at clutch completion (nest age = 9) for mallard, 0.2% (0.1%, 0.3%) on the day the first egg was laid (nest age = 1) to 38% (35%, 41%) at clutch completion (nest age = 10) for gadwall, and 0.2% (0.1%, 0.5%) on the day the first egg was laid (nest age = 1) to 41% (35%, 47%) at clutch completion (nest age = 10) for cinnamon teal.

#### Egg-laying stage vs. incubation stage

Overall nest attendance was 55%, 60%, and 38% lower during the egg laying stage than the incubation stage for mallard, gadwall, and cinnamon teal, respectively (Table 1). Similarly, incubation constancy was 74%, 75%, and 59% lower during the egg-laying stage than during the incubation stage for mallard, gadwall, and cinnamon teal, respectively (Table 1). During the egg-laying stage, mallard, gadwall, and cinnamon teal nests reached nest-specific incubation temperatures 56%, 62%, and 65% of the time that nests were attended, respectively. After clutch completion, mallard, gadwall, and cinnamon teal nests reached incubation temperatures ≥97% of the time that nests were attended.

**Table 1:** Least squares mean (95% confidence interval) nest attendance, incubation constancy, and incubation bout duration during the egg-laying stage and incubation stage for mallard, gadwall, and cinnamon teal nesting in Suisun Marsh, California, 2015–2019.

	Mallard	Gadwall	Cinnamon teal
Nest attendance			
Egg-laying	37.2% (35.4–39.1%)	33.0% (30.6–35.5%)	50.1% (44.8–55.5%)
Incubation	82.4% (82.0–82.8%)	83.1% (82.7–83.5%)	80.8% (79.4–82.2%)
Incubation constancy			
Egg-laying	20.8% (19.2–22.4%)	20.3% (18.2–22.5%)	32.7% (27.2–38.8%)
Incubation	79.9% (79.4–80.5%)	80.7% (80.1–81.2%)	79.6% (77.6–81.4%)
Incubation bout duration (minutes)			
Egg-laying	377 (349–407)	350 (309–395)	559 (460–666)
Incubation	636 (620–652)	779 (759–800)	347 (328–366)

#### Clutch size effects

**Fig 3:**
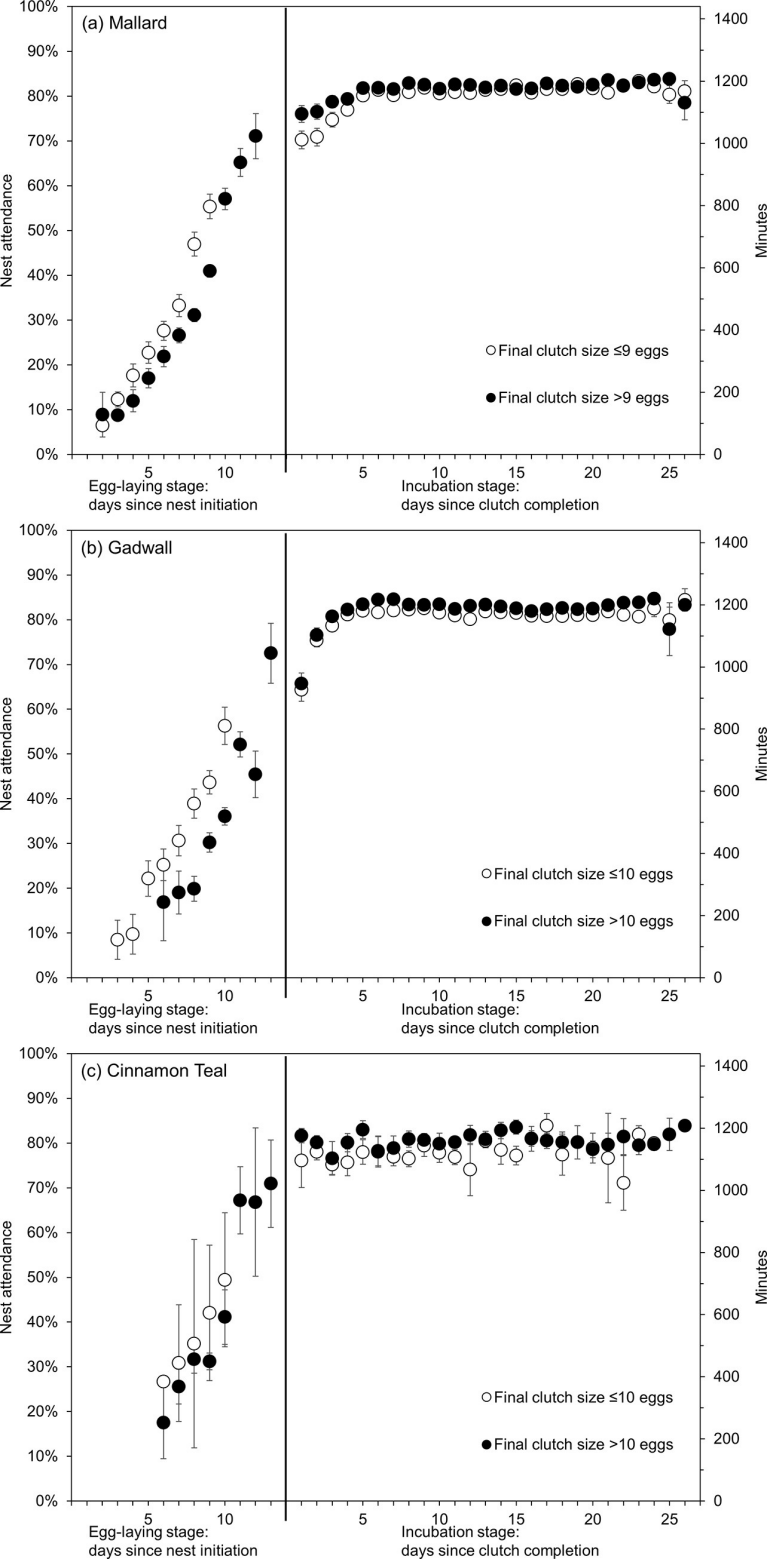
Raw data means ± standard error nest attendance (left y-axis) and corresponding minutes in the day (right y-axis) by (a) mallard, (b) gadwall, and (c) cinnamon teal hens by nest age during the egg-laying stage and incubation stage in Suisun Marsh, California, 2015–2019. Nests are separated into ‘small’ (≤median final clutch size; white circles) and ‘large’ (>median final clutch size; black circles) clutches depending on their final clutch sizes when egg-laying was complete. Nest age = 1 during the egg-laying stage denotes the day the first egg was laid. Nest age = 1 during the incubation stage denotes the first day after clutch completion.

During the egg-laying stage, hens with smaller final clutch sizes showed greater daily nest attendance by nest age than hens with larger final clutch sizes in all three species (Fig 3). Mean (95% confidence interval) model-predicted values show that through the first 8 days, mallard hens at nests with an eventual final clutch size of 8 eggs spent 20% (18%, 22%) of the time on the nest, whereas mallard hens at nests with an eventual final clutch size of 10 eggs spent 15% (14%, 17%) of the time on the nest. Similarly, through the first 9 days, gadwall hens at nests with an eventual final clutch size of 9 eggs spent 19% (17%, 22%) of the time on the nest, whereas gadwall hens at nests with an eventual final clutch size of 11 eggs spent only 14% (12%, 15%) of the time on the nest. Conversely, after clutch completion, daily nest attendance was slightly lower among nests with smaller clutches than larger clutches (Fig 3). For mallard, model-predicted values showed that during the 25 days after clutch completion, mallard hens with a final clutch size of 10 eggs spent an average of 83% (82%, 84%) of the time on the nest, whereas mallard hens with a final clutch of 8 eggs spent an average of 81% (80%, 82%) of the time on the nest. Gadwall hens with a final clutch size of 11 eggs spent an average of 83% (82%, 84%) of the time on the nest during the 25 days after clutch completion, whereas gadwall hens with a final clutch size of 9 eggs spent 81% (80%, 82%) of the time on the nest. When considering incubation constancy, results were similar; nests with smaller final clutch sizes exhibited greater incubation constancy by nest age during the egg-laying stage but lower incubation constancy by nest age during the incubation stage after clutch completion. Through the first 8 days, incubation constancy of mallard nests with eventual final clutch sizes of 8 eggs was 11% (10%, 13%) and 10 eggs was 6% (5%, 6%). Similarly, through the first 9 days, incubation constancy of gadwall nests with eventual final clutch sizes of 9 eggs was 12% (10%, 14%) and 11 eggs was 7% (6%, 8%). After clutch completion, incubation constancy of mallard nests with final clutch sizes of 8 eggs was 78% (76%, 79%) and 10 eggs was 80% (80%, 81%), and incubation constancy of gadwall nests with final clutch sizes of 9 eggs was 78% (76%, 79%) and 11 eggs was 81% (79%, 82%).

#### Nest initiation date effects

Nest initiation date had a moderate influence on nest attendance and incubation constancy and the direction of the effect varied between mallard and gadwall. For mallard, nest attendance was lowest early in the season and increased as the season progressed. Model-predicted mean nest attendance for a 9-egg mallard clutch over the 35-day life of the nest was 17% (11%, 24%) greater for a nest initiated on the median nest initiation date (day of year 115) compared to a nest initiated on the earliest nest initiation date (day of year 63), whereas it was only 1% (-1%, 3%) greater for a nest initiated on the latest nest initiation date (day of year 168) compared to a nest initiated on the median nest initiation date. In contrast, gadwall nest attendance was highest early in the season, decreased as the season progressed, and then increased again late in the season. Model-predicted mean nest attendance for a 10-egg gadwall clutch over the 35-day life of the nest was 7% (3%, 9%) greater for a nest initiated on the earliest nest initiation date (day of year 83) compared to a nest initiated on the median nest initiation date (day of year 129) and 3% (1%, 6%) greater for a nest initiated on the latest nest initiation date (day of year 184) compared to a nest initiated on the median nest initiation date.

### Influences of time of day on nest attendance and incubation constancy

**Fig 4:**
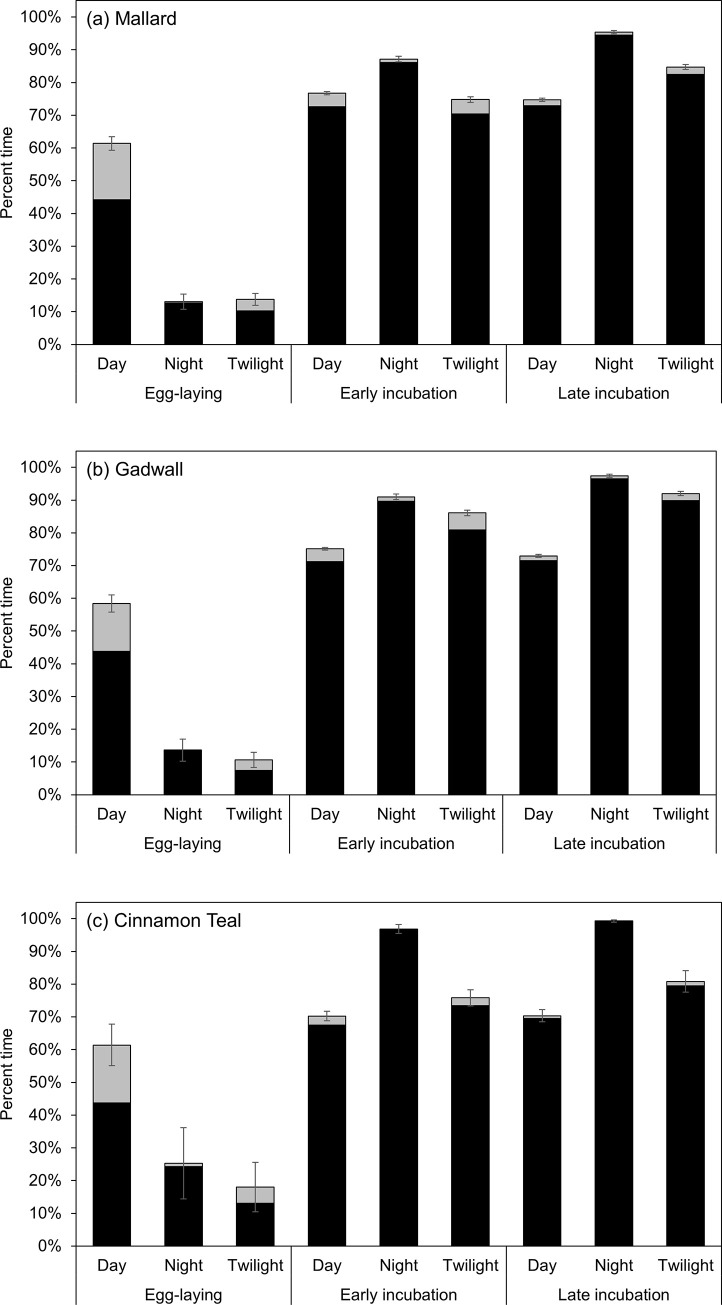
Raw data means (95% confidence limits) of nest attendance (black + gray bars) and incubation constancy (black bars only) for (a) mallard, (b) gadwall, and (c) cinnamon teal nests during daylight hours (solar zenith angle<90°), nighttime hours (solar zenith angle>102°), and nautical twilight hours (solar zenith angle≥90° and ≤102°) during the egg-laying stage, early incubation (1–13 days after clutch completion) and late incubation (14–26 days after clutch completion) in Suisun Marsh, California, 2015–2019.

During the egg-laying stage, nest attendance was much greater during the day (raw data mean mallard: 61%, gadwall: 58%, cinnamon teal: 61%) than at night (mallard: 13%, gadwall: 14%, cinnamon teal: 25%) or during twilight (mallard: 14%, gadwall: 11%, cinnamon teal: 18%; Fig 4). During early incubation (1–13 days after clutch completion), nest attendance increased relative to the egg-laying stage at all three times of day and was greater at night (mallard: 87%, gadwall: 91%, cinnamon teal: 97%) than during the day (mallard: 77%, gadwall: 75%, cinnamon teal: 70%). During late incubation (14–26 days after clutch completion), nest attendance increased relative to early incubation at night (mallard: 95%, gadwall: 97%, cinnamon teal: 99%) and during twilight (mallard: 85%, gadwall: 92%, cinnamon teal: 81%) but not during the day (mallard: 75%, gadwall: 73%, cinnamon teal: 70%; Fig 4).

When hens were present during the egg-laying stage, mallard, gadwall, and cinnamon teal nests reached nest-specific incubation temperatures 72–75%, 97–98%, and 69–74% of the time during the day, night, and twilight, respectively (Fig 4). During the early and late incubation stages, nests reached nest-specific incubation temperatures ≥94% of the time for all time periods in all three species (Fig 4).

### Variation in nest temperature

**Fig 5:**
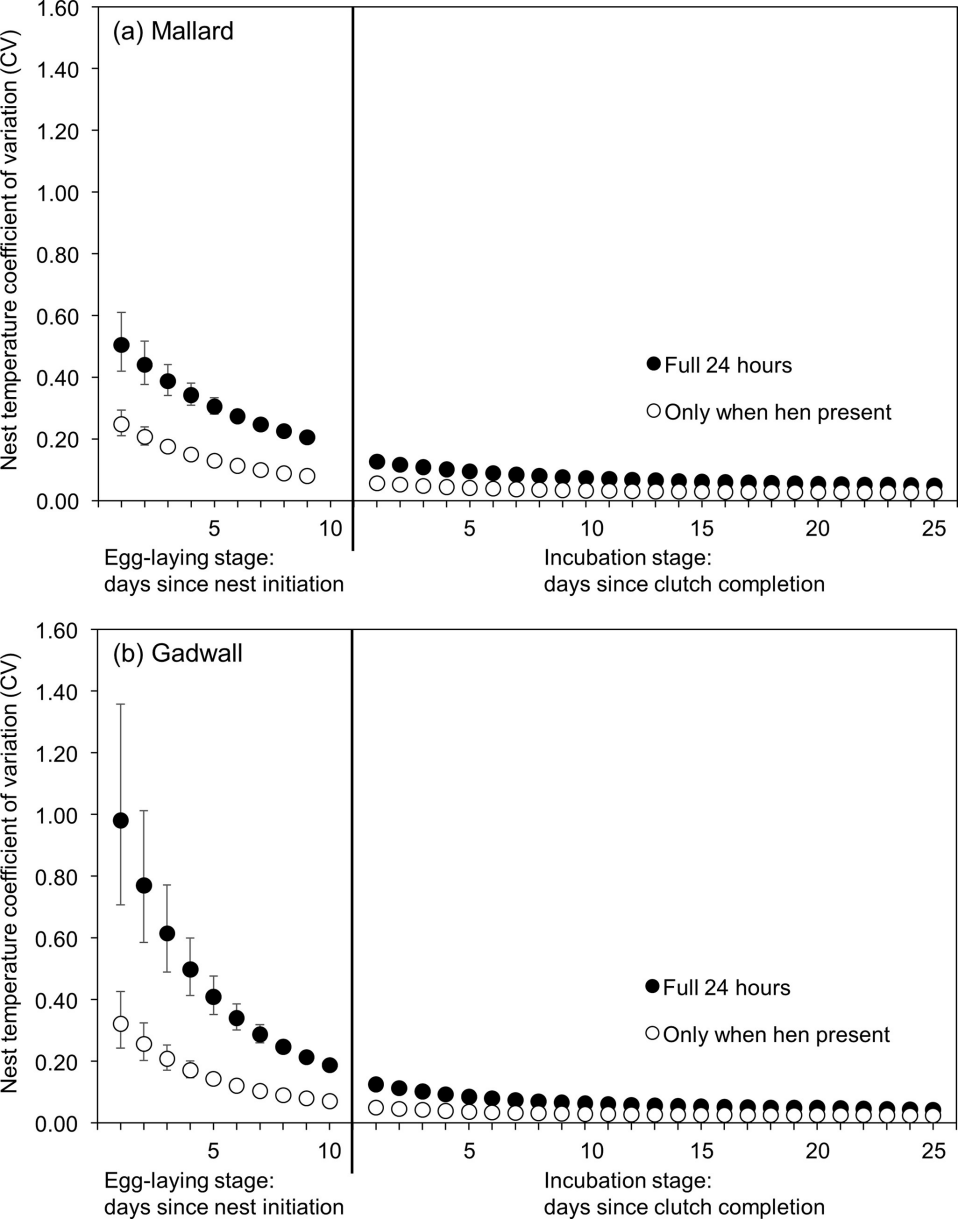
Model-averaged predicted coefficients of variation (CV; standard deviation/mean) for nest temperature over 24 hours (black circles) and nest temperature only when the hen was present at the nest (white circles) by nest age for (a) mallard and (b) gadwall nests in Suisun Marsh, California, 2015–2019. Predictions were made at the median final clutch size (mallard: 9 eggs, gadwall: 10 eggs) and median nest initiation date as day of year (mallard: 115, gadwall: 129).

For both mallard and gadwall, the variables nest stage, nest age, final clutch size, and nest initiation date, were all important predictors of daily variation in nest temperature (S3 Table). In addition, the interactions nest stage×final clutch size and final clutch size×nest initiation date were present in the top-ranked model in both species. Model-averaged predictions showed that daily variation in nest temperature was greater, indicating less consistent nest temperatures, during the egg-laying stage than during the incubation stage and variation in nest temperature decreased with nest age (Fig 5). Variation in nest temperature decreased sharply after clutch completion as nest attendance and incubation constancy increased, leading to more consistent nest temperatures. For a 9-egg mallard clutch, model-predicted variation in nest temperature decreased by 38% (from 0.206 to 0.127) between the day of and the day after clutch completion. Similarly, for a 10-egg gadwall clutch, variation in nest temperature decreased by 33% (from 0.187 to 0.126) between the day of and the day after clutch completion.

When considering variation in nest temperature only during the times the hen was attending the nest, model selection results were similar (S4 Table). Model-averaged predictions showed that variation in nest temperature during hen attendance decreased sharply after clutch completion, decreasing 29% (from 0.080 to 0.057) for a 9-egg mallard clutch and 29% (from 0.071 to 0.051) for a 10-egg gadwall clutch between the day of and the day after clutch completion (Fig 5).

Although overall variation in nest temperature decreased with nest age during both the egg-laying stage and incubation stage, the rate of decrease was much greater during egg-laying (Fig 5). During the 9-day (mallard) and 10-day (gadwall) median egg-laying stage, variation in nest temperature decreased 59% for mallard and 81% for gadwall, whereas during the 25 days after clutch completion variation in nest temperature, already low relative to the egg laying stage, decreased 62% for mallard and 67% for gadwall. Examining variation in nest temperature only when the hen was attending the nest yielded similar results. Variation in nest temperature when the hen was present decreased 68% for mallard and 78% for gadwall during the egg-laying stage and 53% for both species during the 25-day incubation stage after clutch completion.

The influences of final clutch size and nest initiation date on variation in nest temperature among mallard and gadwall nests were similar to their influences on nest attendance and incubation constancy. During egg-laying, nests with larger final clutch sizes exhibited greater variation in daily nest temperature by nest age than did nests with smaller final clutch sizes. During the first 8 days of egg-laying, variation in nest temperature averaged 6% higher for a mallard nest with an eventual final clutch size of 10 eggs (0.352) compared to a mallard nest with an eventual final clutch size of 8 eggs (0.331). Similarly, during the first 9 days of egg-laying, variation in nest temperature averaged 18% higher for a gadwall nest with an eventual final clutch size of 11 eggs (0.527) compared to a gadwall nest with an eventual final clutch size of 9 eggs (0.445). Conversely, during the incubation period after clutch completion, variation in nest temperature was 15% higher among mallard nests with a final clutch size of 8 eggs (0.080) compared to 10 eggs (0.068) and 11% higher among gadwall nests with a final clutch size of 9 eggs (0.070) compared to 11 eggs (0.062). Finally, daily variation in nest temperature for both species decreased with nest initiation date.

### Incubation bout duration

We identified 19,725 individual incubation bouts (11,323 mallard, 7,124 gadwall, 1,278 cinnamon teal), including 1,403 incubation bouts during the egg-laying stage and 18,322 incubation bouts during the incubation stage after clutch completion.

**Fig 6:**
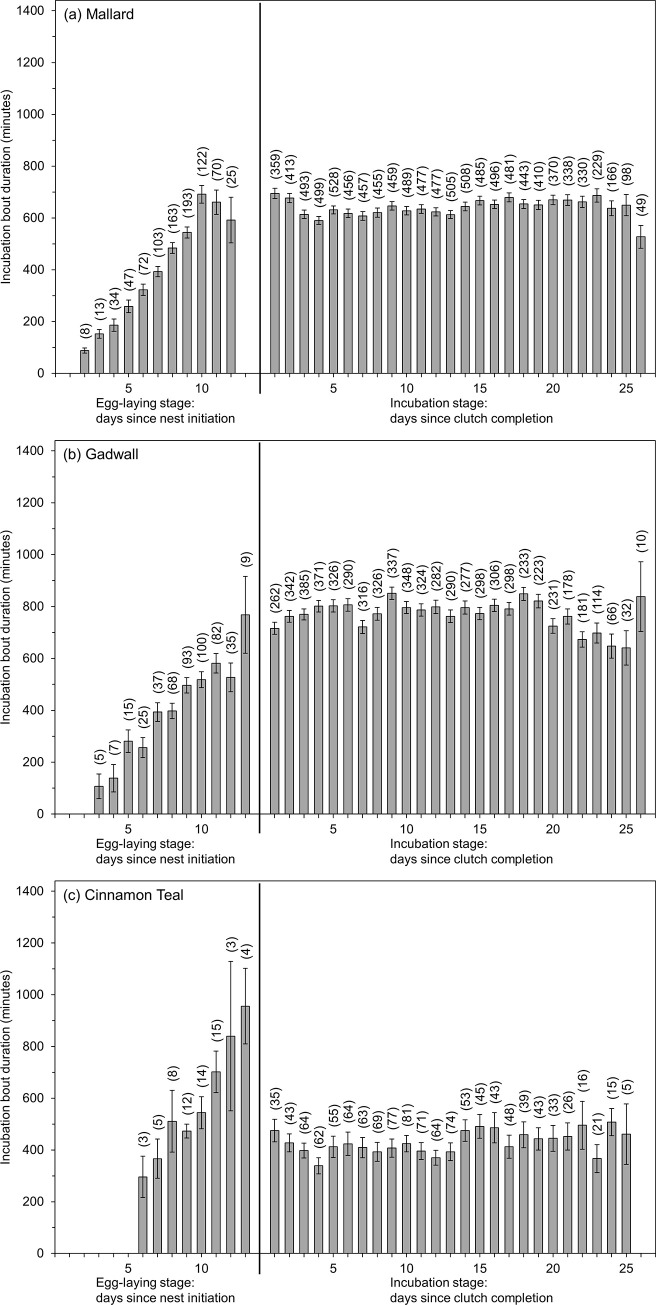
Raw data means ± standard error for incubation bout duration for (a) mallard, (b) gadwall, and (c) cinnamon teal nests by nest age during the egg-laying stage and incubation stage in Suisun Marsh, California, 2015–2019. Sample sizes for each nest age are in parentheses. Nest age = 1 during the egg-laying stage denotes the day the first egg was laid. Nest age = 1 during the incubation stage denotes the first day after clutch completion. The Y-axis is scaled to 1440 minutes, or a full 24-hr day.

All three species exhibited increasing mean incubation bout duration with nest age during the egg-laying stage followed by a more constant mean incubation bout duration during the incubation stage after clutch completion (Fig 6). Raw data means (± SD) reveal that after clutch completion, incubation bout duration was longest for gadwall (778±421 minutes), followed by mallard (641±363 minutes), and then cinnamon teal (421±292 minutes; Fig 6). Incubation bout duration for mallard and gadwall exhibited a bimodal distribution. Mallard bout duration peaked at ~600 minutes with a smaller second peak at ~1200 minutes. The distribution of gadwall incubation bout duration was the inverse, with a large peak at ~1200 minutes and a smaller peak at ~600 minutes. These 2 peaks in incubation bout duration corresponded to the number of incubation recesses hens took in a day. When hens took one recess (42% of mallard nest-days, 64% of gadwall nest-days; [18]), the incubation bout durations were closer to 1200 minutes, whereas when hens took 2 recesses, the incubation bout durations were each closer to 600 minutes. In contrast, cinnamon teal hens took 2–3 incubation recesses per day and incubation bout durations were shorter, with durations peaking at around 400 minutes.

**Fig 7:**
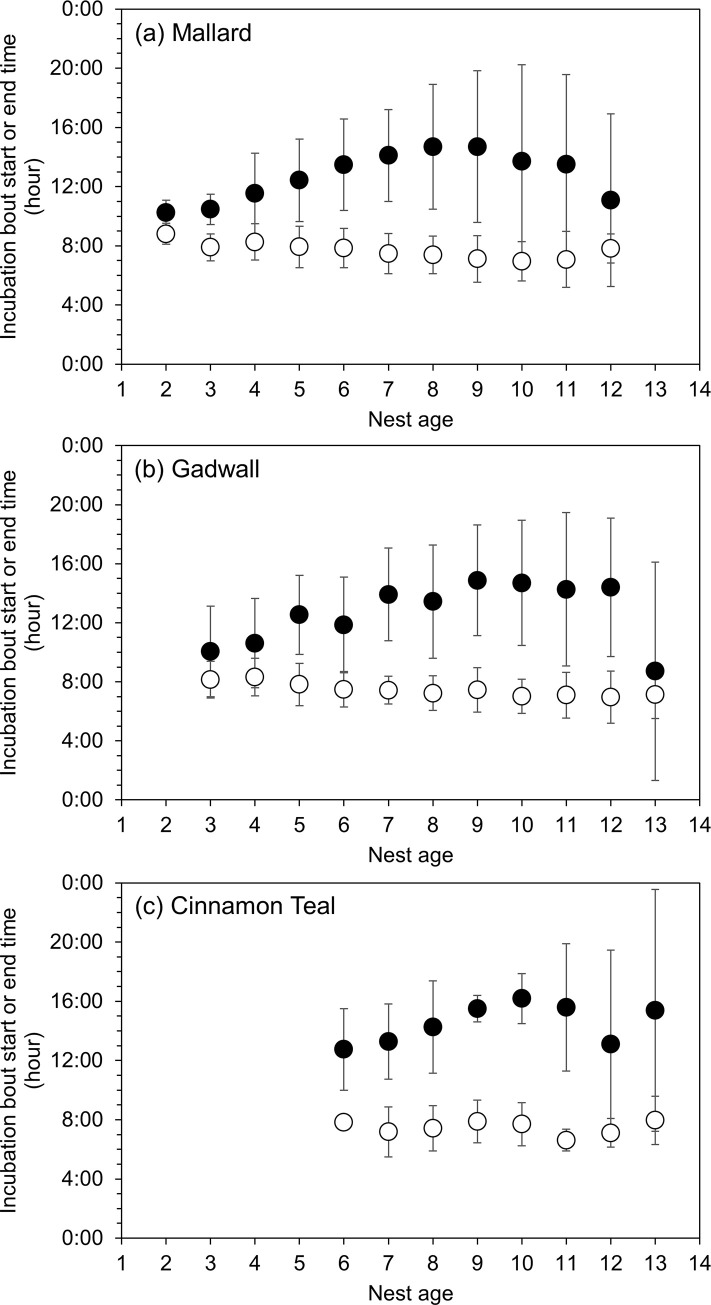
Raw data means ± standard deviation morning incubation bout start time (white circles) and end times (black circles) by nest age during the egg-laying stage for (a) mallard, (b) gadwall, and (c) cinnamon teal in Suisun Marsh, California, 2015–2019. Nest age = 1 denotes the day the first egg was laid.

**Fig 8:**
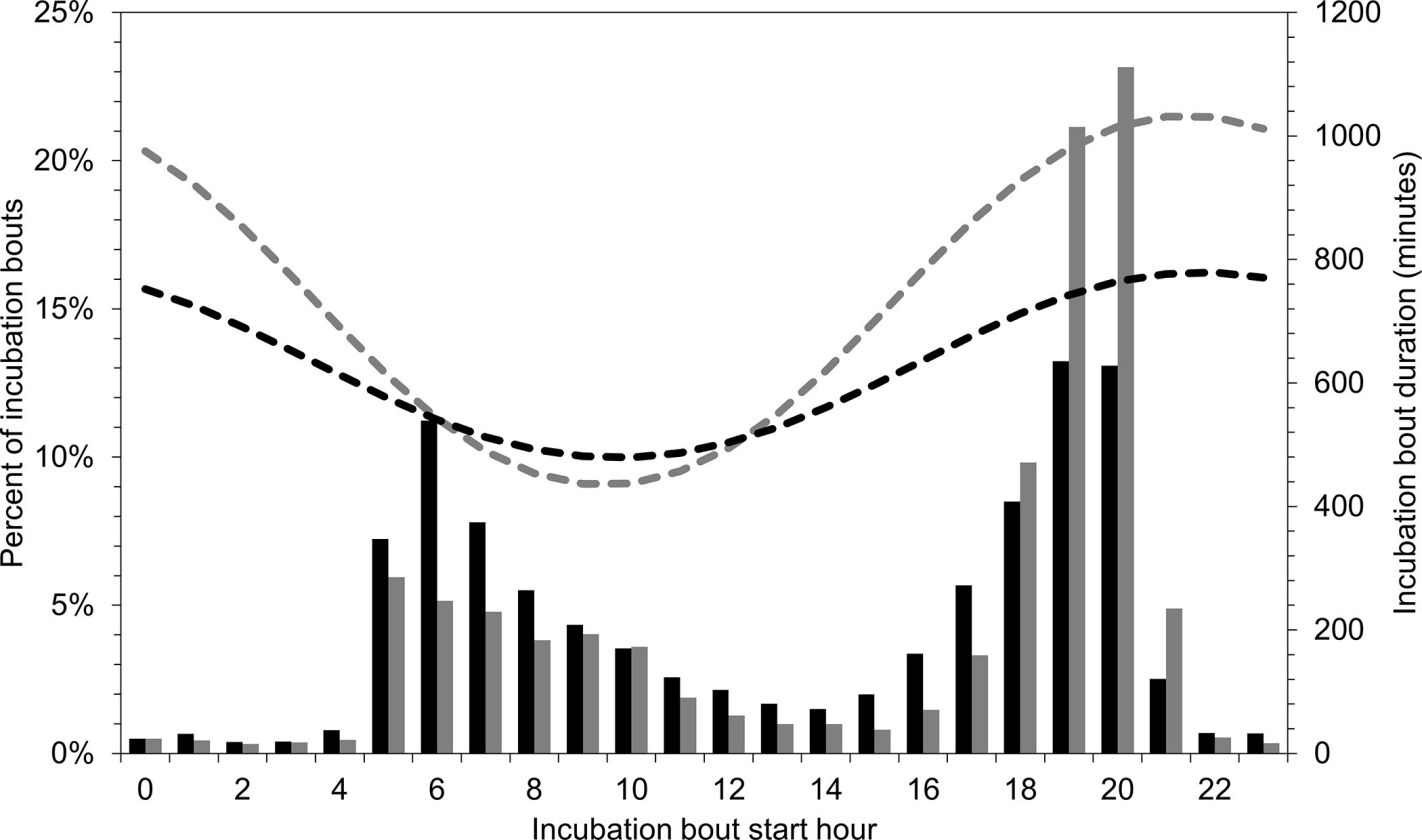
The percentage of incubation bouts during the incubation stage after clutch completion by the hour in which they began for mallard (black bars) and gadwall (gray bars), and mean model-predicted incubation bout duration by the hour in which the incubation bout began for mallard (black dashed line) and gadwall (gray dashed line) nests in Suisun Marsh, California, 2015–2019. Predictions were made at nest age = 15 days, the median final clutch size (mallard: 9 eggs, gadwall: 10 eggs), and the median nest initiation date as day of year (mallard: 115, gadwall: 129).

The time of day when hens initiated incubation bouts varied between the egg-laying stage and the incubation stage and by species. During egg-laying, all three species initiated almost all incubation bouts in the early morning hours (05:00–10:00), accounting for 86% of mallard bouts, 87% of gadwall bouts, and 81% of cinnamon teal bouts. As egg-laying progressed, hens initiated incubation bouts slightly earlier each day and ended incubation bouts later in the day (Fig 7). During the incubation stage after clutch completion, mallard hens most often initiated incubation bouts in the early morning (05:00–10:00; 40% of bouts) or early evening (17:00–20:00; 41% of bouts). Similarly, cinnamon teal hens most often initiated incubation bouts in the early morning (05:00–10:00; 39% of bouts) or late afternoon and early evening (16:00–20:00, 47% of bouts). In contrast, only 27% of gadwall incubation bouts during the incubation stage were initiated between 05:00 and 10:00, whereas 62% of bouts were initiated between 17:00 and 21:00 (Fig 8).

For mallard and gadwall, the variables nest stage, nest age, final clutch size, and the hour and ambient temperature when the incubation bout started were important predictors of bout duration and for cinnamon teal, the top-ranked model included nest stage, a linear trend for nest age, and the interaction nest stage×nest age (S5 Table). Overall duration of individual incubation bouts was 41% and 55% shorter during the egg-laying stage than during the incubation stage for mallard and gadwall, respectively but for cinnamon teal, was 61% longer during the egg-laying stage than during the incubation stage (Table 1). Mean (95% confidence interval) model-predicted duration for incubation bouts starting during the 06:00 hour increased from 67 (25, 174) minutes on the day of nest initiation (nest age = 1) to 508 (246, 851) minutes on the day of clutch completion (nest age = 9) for mallard, and increased from 99 (28, 312) minutes on the day of nest initiation (nest age = 1) to 439 (178, 832) minutes on the day of clutch completion (nest age = 10) for gadwall. After clutch completion, incubation bouts starting during the 06:00 hour averaged 551 (275, 891) minutes for mallard and 512 (219, 906) minutes for gadwall, whereas incubation bouts starting during the 20:00 hour averaged 775 (443, 1085) for mallard, and 986 (597, 1252) for gadwall.

The hour and ambient temperature when the incubation bout began had a large effect on incubation bout duration. Incubation bout duration decreased as ambient temperature at the beginning of the incubation bout increased and was longest for bouts beginning during the evening through early morning (18:00–02:00) and was shortest for bouts beginning during the late morning and early afternoon (07:00–13:00; Fig 8).

Finally, for both mallard and gadwall, nests with larger final clutch sizes exhibited incubation bout durations that were shorter during the egg-laying stage but longer during the incubation stage than nests with smaller final clutch sizes. For example, during the first 8 days of egg-laying, mallard incubation bouts starting during the 06:00 hour, on average, were 24% shorter among mallard nests with an eventual 10-egg final clutch size (178 minutes) than mallard nests with an eventual 8-egg final clutch size (234 minutes), but after clutch completion, incubation bouts were 10% longer among mallard nests with a 10-egg final clutch size (579 minutes) than mallard nests with an 8-egg final clutch size (522 minutes). Similarly, during the first 9 days of egg-laying, gadwall incubation bouts starting during the 06:00 hour, on average, were 14% shorter among gadwall nests with an eventual 11-egg final clutch size (202 minutes) than gadwall nests with an eventual 9-egg final clutch size (236 minutes), but after clutch completion, incubation bouts were 8% longer among gadwall nests with a 11-egg final clutch size (533 minutes) than gadwall nests with a 9-egg final clutch size (492 minutes).

## Discussion

### Incubation behavior among species

All three dabbling duck species we studied showed similar levels of nest attendance and incubation constancy after clutch completion. Mallard, gadwall, and cinnamon teal nest attendance averaged 82%, 83%, and 81% respectively, and incubation constancy averaged 80%, 81%, and 80% respectively, during the incubation stage after clutch completion. Values for all three species were similar to those observed in some other studies of dabbling ducks, including mallard, gadwall, and cinnamon teal [16,46,47]. Although overall daily nest attendance and incubation constancy were similar among mallard, gadwall, and cinnamon teal, patterns of nest attendance during the day varied by species. Previously Croston et al. [18] reported that, after clutch completion, mallard predominantly took one (42% of nest-days) or 2 (45% of nest-days) incubation recesses each day, whereas gadwall predominantly took only one recess (64% of nest-days) each day. Cinnamon teal typically took 2–3 incubation recesses each day. For all three species, a high proportion of incubation bouts began in the early evening, because hens returned from afternoon recesses to begin long incubation bouts that lasted overnight (Fig 8). Yet, whereas mallard and cinnamon teal often then took an incubation recess in the early morning, gadwall were more likely to forgo this morning incubation recess, resulting in a considerably longer incubation bout stretching from late afternoon or evening until the afternoon of the following day. As a result, average incubation bout duration for gadwall (779 minutes) was 22% longer than for mallard (636 minutes) and 125% longer than for cinnamon teal (347 minutes). Afton [20] and Afton and Paulus [16] proposed that nest attendance is positively correlated with body size and that the frequency of incubation recesses is negatively correlated with body size, because smaller birds may be more nutrient limited than larger birds, necessitating more total time off the nest and more frequent recesses to forage. Our results largely follow this expectation as the larger mallard hens (~900 g) and gadwall hens (~650 g) had higher nest attendance and took fewer recesses than did the much smaller cinnamon teal hens (~350 g).

During the egg-laying stage, all three species typically exhibited one incubation bout each day, which began in the early morning, when hens usually lay a new egg [48–50]. Incubation bout duration, much like overall nest attendance, increased daily during the egg-laying stage. This increase in incubation bout duration with age consisted of hens beginning their incubation bouts slightly earlier in the day and ending their incubation bout later in the day as egg-laying progressed (Fig 7), a result similar to what has been observed in other dabbling ducks [20,29]. By the last few days of egg-laying, average incubation bout duration was closer to what was observed after clutch completion (Fig 6). The exception was cinnamon teal, in which average incubation bout duration was longer before clutch completion (559 minutes) than after clutch completion (347 minutes), because of their more frequent incubation recesses after clutch completion (2–3 per day) compared to mallard and gadwall (1–2 per day). Nevertheless, cinnamon teal overall incubation constancy still increased following clutch completion.

### Onset of incubation

Mallard, gadwall, and cinnamon teal hens all began actively incubating their eggs during the egg-laying stage prior to clutch completion. As soon as the day after the first egg was laid (we had no data on the day the first egg was laid), nests began to reach the same nest-specific temperatures observed during the incubation stage after clutch completion, albeit for a much smaller proportion of the day. Although initially quite low, incubation constancy increased rapidly during egg-laying. Model predictions indicated that for a 9-egg mallard clutch, incubation constancy increased from only 0.3% on the day the first egg was laid to 45% on the day of clutch completion, 8 days later. Similarly, for a 10-egg gadwall clutch, incubation constancy increased from only 0.2% on the day the first egg was laid to 38% on the day of clutch completion, and for a 10-egg cinnamon teal clutch from 0.2% on the day the first egg was laid to 41% on the day of clutch completion, 9 days later. Loos and Rohwer [29] reported similar increases in nest attendance during egg-laying in several prairie-nesting dabbling duck species.

Following clutch completion, incubation constancy increased sharply. Model predictions showed that between the day of and the day after clutch completion, incubation constancy increased from 45% (653 minutes) to 72% (1039 minutes) for a 9-egg mallard clutch, from 38% (549 minutes) to 60% (858 minutes) for a 10-egg gadwall clutch, and from 41% (590 minutes) to 75% (1080 minutes) for a 10-egg cinnamon teal clutch. A similar result was observed in variation (CV) in nest temperature, which decreased by 38% and 33% among mallard and gadwall, respectively, between the day of and the day after clutch completion, indicating a more stable thermal environment with the greater incubation constancy brought about by clutch completion.

The large increases in incubation constancy and large decreases in nest temperature variation observed after clutch completion were due primarily to the onset of overnight incubation. During the egg-laying stage, mallard, gadwall, and cinnamon teal hens spent little time on the nest or incubating eggs at night (Figs 2 and 4), with the little time they did spend occurring mostly in the early evening hours or the early morning hours during a diurnal incubation bout. Although diurnal incubation bouts gradually extended longer into the afternoon and early evening as egg-laying progressed, we did not observe hens incubating for a substantial proportion of the nighttime hours until after clutch completion (Fig 2). Previous studies have reported similar results in mallard [19] and northern shoveler (*Spatula clypeata*; Afton 1980). Thus, clutch completion appears to trigger the onset of overnight incubation, which resulted in a large and immediate increase in incubation constancy and stabilization of the thermal environment of the nest.

### Variation in incubation constancy during egg-laying

Active incubation during the egg-laying stage, as observed in this study, means that eggs laid early in the laying sequence received more incubation time than eggs laid later in the laying sequence. Unequal incubation time could lead to differences in developmental rates among eggs within a clutch, potentially leading to hatching asynchrony [51]. Yet, most waterfowl studies have found that hatching asynchrony is rare, and most eggs within a clutch hatch within 24 hours of one another [16,25]. This has generated other hypotheses to explain how eggs laid later in the laying sequence may catch up to earlier laid eggs in development, such as vocalization from more developed embryos from eggs laid early in the laying sequence stimulating development and hatching of sibling embryos from eggs laid later in the laying sequence [34], differences in the rate of metabolism, and thus development, among eggs in the clutch according to their position in the laying sequence [32], and differential incubation temperature and development among eggs in a clutch depending on their physical position in the nest [24,35,36]. In this study, we observed three factors that likely limit incubation time differences, and therefore differences in development rate among eggs within a clutch.

First, nest attendance and incubation constancy increased more slowly during the egg-laying stage among nests with larger eventual final clutch sizes than among nests with smaller eventual final clutch sizes (Fig 3), which reduced the difference in incubation time among individual eggs in the same clutch. Similarly, great tit (*Parus major*) females with larger clutch sizes delayed full nocturnal incubation and partial diurnal incubation during egg-laying compared to females with smaller clutch sizes [52]. In contrast, clutch size had no effect on nest attentiveness during egg-laying in the American coot (*Fulica americana*) [53] and nest attendance during egg-laying was actually greater in two-egg versus three-egg herring gull (*Larus argentatus*) clutches [54]. Among ducks, reduced incubation time during the egg-laying stage for larger clutches has also been observed in blue-winged teal (*Spatula discors*) and northern shoveler [29]. In our study, on day 5 after nest initiation (nest age = 5), incubation constancy of a gadwall nest with an eventual final clutch size of 9 eggs was predicted to be 7% (94 minutes), whereas for a gadwall nest with an eventual final clutch size of 11 eggs, incubation constancy was predicted to be only 4% (50 minutes). Because both nests in this example would have 5 eggs in the nest 5 days after nest initiation, this result is not due to differences in the number of eggs in the nest. Rather, differences in incubation constancy during egg-laying were more related to the number of eggs remaining to be laid, and likely correspond to differences in concentrations of parental care hormones such as prolactin, which have been observed to be lower by nest age among females that laid larger clutches [55].

Larger clutch sizes may be more susceptible to differential embryonic development, and therefore hatching asynchrony. In wood ducks, greater developmental asynchrony and reduced hatching success was observed in nests with larger clutches, indicating a potential cost of incubation during egg-laying, at least among larger clutches [51]. In our study, the slower increase (shallower slope) in incubation constancy with nest age during the egg-laying stage of nests with larger eventual final clutch sizes reduced the potential difference in total incubation time among eggs in the clutch. For example, model predictions show that during the egg-laying stage the first egg laid of an eventual 9-egg gadwall clutch would receive 1542 minutes of total incubation time (day 1 –day 9), whereas the 8^th^ laid egg would receive only 928 minutes of total incubation time (day 8 –day 9), a difference of 614 minutes. In contrast, the first egg laid of an eventual 11-egg gadwall clutch would receive 1892 minutes of incubation time (day 1 –day 11; even with the shallower slope, more time overall due to 2 extra days of egg-laying compared to the 9-egg clutch), whereas the 10^th^ egg laid would receive only 988 minutes of incubation time (day 10 –day 11), a difference of 904 minutes. However, if the slope for the increase in incubation constancy of the gadwall nest with an eventual 11-egg final clutch size was the same as that of the nest with an eventual 9-egg final clutch size, the difference in total incubation time between the first and 10^th^ egg laid in the clutch would have been 1542 minutes, or 638 minutes more than the 904 minutes we observed. Thus, the slower increase in incubation constancy during egg-laying observed among nests with larger eventual final clutch sizes reduced differences in total incubation time among eggs that was brought about by the longer egg-laying stage necessary to lay a larger clutch.

Second, the total amount of time eggs reached nest-specific incubation temperatures (incubation constancy) was lower during the egg-laying stage compared to the incubation stage after clutch completion. During egg-laying mallard, gadwall and cinnamon teal nests reached incubation temperatures only 56%, 62%, and 65% of the time that hens were attending the nest, whereas after clutch completion, nests of all three species reached incubation temperatures ≥97% of the time hens attended nests. A similar relationship was observed in mountain bluebirds (*Sialia currucoides*), where the amount of heat applied to eggs increased as egg-laying progressed and was typically greater after clutch completion [56]. Wood duck nests also exhibited lower temperatures when the hen was present during the first few days of egg-laying compared to later during the egg-laying stage [57]. Relatedly, variation in daily nest temperature dropped considerably after clutch completion because greater nest attendance created a more stable thermal environment for the eggs (Fig 5). But variation in nest temperature only during the times the hen was attending the nest also declined after clutch completion. Taken together, these results indicate that hens may reduce developmental asynchrony among eggs not only by limiting their time spent on the nest during egg-laying, but also by providing a less effective thermal environment for embryonic development when they are on the nest.

Third, overnight incubation did not begin until after the clutch had been completed. Prior to clutch completion, nest attendance and incubation constancy gradually increased daily, as hens ended their incubation bouts later in the day as egg-laying progressed. But it was only at the time of clutch completion that overnight incubation began, resulting in a large and immediate increase of several hours (~21:00–04:00) to daily incubation constancy. This result is perhaps explained by the risks of incubation to the hen compared to the changing expected value of the current clutch [58,59]. In Suisun Marsh, as in other nesting areas, nocturnal mammals are the primary egg predators of dabbling duck nests and mammals sometimes kill incubating hens [60,61]. Thus, incubating at night can be riskier for the hen than incubating during the day. At the same time, temperatures are relatively mild at our study site, with average lows (~10°C) above what is likely to negatively affect eggs due to a lack of incubation at night [3]. During the egg-laying stage, the expected value of the partially laid clutch is low relative to a fully laid clutch during the incubation stage. Incubating at night, therefore, may be too risky for the hen relative to the expected benefit of the offspring until after clutch completion, when the expected value of the reproductive effort increases. By delaying overnight incubation until after clutch completion, hens avoid this increased risk to themselves, and consequently, limit incubation time when it would apply unequally among eggs (egg-laying stage) based on egg position in the laying sequence, and potentially exacerbate hatching asynchrony.

In summary, we observed similar rates of nest attendance and incubation constancy among mallard, gadwall, and cinnamon teal during the egg-laying stage and incubation stage. However, despite similarities in nest attendance, we observed notable species differences in daily incubation rhythms, with gadwall predominantly taking one incubation recess, mallard taking 1–2 recesses, and cinnamon teal taking 2–3 recesses per day. The result was that average incubation bout duration was longest in gadwall, followed by mallard, and then cinnamon teal. We also observed clutch size differences in nest attendance and incubation constancy in all three species, with larger clutches exhibiting lower nest attendance and shorter incubation bouts during the egg-laying stage than smaller clutches, but greater nest attendance and longer incubation bouts during the incubation stage. Moreover, during the incubation stage, hens heated their eggs to average nest-specific incubation temperatures almost all of the time (≥97%) they were on the nest, whereas during the egg-laying stage, hens heated eggs to incubation temperatures only 56–65% of the time they were on the nest. These observations have important implications for dabbling duck egg development and reproductive success, including mitigating differences in total incubation time among eggs in the clutch to promote hatching synchrony and reducing the risk of nest depredation.

## Supporting information

S1 Table Model selection results for linear mixed models of daily nest attendance (percent time spent on the nest) for (A) Mallard, (B) Gadwall, and (C) cinnamon teal in Suisun Marsh, California, 2015–2019. (DOCX)

S2 Table Model selection results for linear mixed models of daily incubation constancy for (A) Mallard, (B) Gadwall, and (C) Cinnamon teal in Suisun Marsh, California, 2015–2019. (DOCX)

S3 Table Model selection results for linear mixed models of daily nest temperature coefficient of variation (CV) over 24 hours for (A) Mallard and (B) Gadwall in Suisun Marsh, California, 2015–2019. (DOCX)

S4 Table Model selection results for linear mixed models of daily nest temperature coefficient of variation (CV) when the hen was in attendance for (A) Mallard and (B) Gadwall in Suisun Marsh, California, 2015–2019. (DOCX)

S5 Table Model selection results for linear mixed models of incubation bout duration for (A) Mallard, (B) Gadwall, and (C) Cinnamon teal in Suisun Marsh, California, 2015–2019. (DOCX)
