# Supplementary material for: Nest attendance, incubation constancy, and onset of incubation in dabbling ducks
Source: PLoS One. 2023 May 19;18(5):e0286151. doi: 10.1371/journal.pone.0286151 (PMC10198486; doi:10.1371/journal.pone.0286151)
Supplement: S3 Table — (DOCX) [file pone.0286151.s003.docx]

**S3 Table.** Model selection results for linear mixed models of daily nest temperature coefficient of variation (CV) over 24 hours for (**A**) Mallard and (**B**) Gadwall in Suisun Marsh, California, 2015-2019. All models include nest identification as a random effect to account for non-independence of repeated measures within nests. Models in the table include the top-ranked models representing 0.99 cumulative model weights, the null model (random effect and error only), and in bold, the top model and models that are the same as the top model but with one of the variables removed. Nest Stage is a categorical variable that includes the egg-laying stage and the incubation stage after the clutch has been completed. Final Clutch Size is the clutch size after all eggs have been laid. NID = Nest initiation date. Variables with a superscript of 2 or 3 indicate quadratic and cubic trends, respectively.

| Model | *K*^a^ | -2LogL | AIC_c_^b^ | ΔAIC_c_^c^ | *w_i_*^d^ | Evidence ratio^e^ |
| --- | --- | --- | --- | --- | --- | --- |
| (**A**) Mallard |  |  |  |  |  |  |
| **Stage + Final Clutch Size + Nest Age^3^ + NID + Stage×Final Clutch Size + Final Clutch Size×NID** | **11** | **7158.40** | **7180.44** | **0.00** | **0.48** | **1.00** |
| Stage + Final Clutch Size + Nest Age^3^ + NID^2^ + Stage×Final Clutch Size | 11 | 7159.91 | 7181.95 | 1.51 | 0.22 | 2.12 |
| Stage + Final Clutch Size + Nest Age^3^ + NID^2^ + Stage×Final Clutch Size + Final Clutch Size×NID^2^ | 13 | 7157.21 | 7183.27 | 2.82 | 0.12 | 4.10 |
| **Stage + Final Clutch Size + Nest Age^3^ + NID + Stage×Final Clutch Size** | **10** | **7163.66** | **7183.69** | **3.25** | **0.09** | **5.08** |
| Stage + Final Clutch Size + Nest Age^2^ + NID + Stage×Final Clutch Size + Final Clutch Size×NID | 10 | 7165.15 | 7185.18 | 4.74 | 0.04 | 10.70 |
| Stage + Final Clutch Size + Nest Age^2^ + NID^2^ + Stage×Final Clutch Size | 10 | 7166.72 | 7186.75 | 6.31 | 0.02 | 23.45 |
| Stage + Final Clutch Size + Nest Age^2^ + NID^2^ + Stage×Final Clutch Size + Final Clutch Size×NID^2^ | 12 | 7163.79 | 7187.84 | 7.40 | 0.01 | 40.37 |
| Stage + Final Clutch Size + Nest Age^2^ + NID + Stage×Final Clutch Size | 9 | 7170.68 | 7188.71 | 8.27 | 0.01 | 62.38 |
| **Stage + Final Clutch Size + Nest Age^3^ + NID + Final Clutch Size×NID** | **10** | **7171.64** | **7191.67** | **11.23** | **0.00** | **274.29** |
| **Stage + Nest Age^3^ + NID** | **8** | **7192.14** | **7208.16** | **27.72** | **0.00** | **1.04×10^6^** |
| **Stage + Final Clutch Size + Nest Age^3^ + Stage×Final Clutch Size** | **9** | **7235.69** | **7253.72** | **73.28** | **0.00** | **8.16×10^15^** |
| **Final Clutch Size + Nest Age^3^ + NID + Final Clutch Size×NID** | **9** | **7358.51** | **7376.53** | **196.09** | **0.00** | **3.81×10^42^** |
| **Stage + Final Clutch Size + NID + Stage×Final Clutch Size + Final Clutch Size×NID** | **8** | **7864.63** | **7880.65** | **700.21** | **0.00** | **1.12×10^152^** |
| Null | 3 | 8604.17 | 8610.17 | 1429.73 | 0.00 | >6.45×10^304^ |
| (**B**) Gadwall |  |  |  |  |  |  |
| **Stage + Final Clutch Size + Nest Age^3^ + NID^2^ + Stage×Final Clutch Size + Final Clutch Size×NID^2^** | **13** | **6169.55** | **6195.61** | **0.00** | **0.48** | **1.00** |
| Stage + Final Clutch Size + Nest Age^3^ + NID + Stage×Final Clutch Size | 10 | 6177.48 | 6197.52 | 1.91 | 0.19 | 2.60 |
| **Stage + Final Clutch Size + Nest Age^3^ + NID^2^ + Stage×Final Clutch Size** | **11** | **6175.51** | **6197.56** | **1.95** | **0.18** | **2.65** |
| Stage + Final Clutch Size + Nest Age^3^ + NID + Stage×Final Clutch Size + Final Clutch Size×NID | 11 | 6176.19 | 6198.24 | 2.63 | 0.13 | 3.72 |
| **Stage + Nest Age^3^ + NID^2^** | **9** | **6187.26** | **6205.30** | **9.68** | **0.00** | **126.75** |
| Stage + Nest Age^3^ + NID | 8 | 6189.31 | 6205.33 | 9.72 | 0.00 | 128.99 |
| **Stage + Final Clutch Size + Nest Age^3^ + NID^2^ + Final Clutch Size×NID^2^** | **12** | **6181.41** | **6205.47** | **9.86** | **0.00** | **138.19** |
| **Stage + Final Clutch Size + Nest Age^3^ + Stage×Final Clutch Size** | **9** | **6192.43** | **6210.47** | **14.85** | **0.00** | **1680.81** |
| **Final Clutch Size + Nest Age^3^ + NID^2^ + Final Clutch Size×NID^2^** | **11** | **6250.70** | **6272.75** | **77.14** | **0.00** | **5.63×10^16^** |
| **Stage + Final Clutch Size + NID^2^ + Stage×Final Clutch Size + Final Clutch Size×NID^2^** | **10** | **6777.83** | **6797.87** | **602.25** | **0.00** | **6.00×10^130^** |
| Null | 3 | 7138.50 | 7144.50 | 948.89 | 0.00 | 1.12×10^206^ |

^a^ The number of parameters in the model.

^b^ Akaike’s Information Criterion corrected for small sample size.

^c^ The difference in the AIC_c_ values of the current model and the model with the lowest AIC_c_.

^d^ Akaike model weight. The likelihood of the model given the data, relative to other models in the candidate set.

^e^ The weight of evidence that the model with the lowest AIC_c_ value is better than the current model.
