# Supplementary material for: Nest attendance, incubation constancy, and onset of incubation in dabbling ducks
Source: PLoS One. 2023 May 19;18(5):e0286151. doi: 10.1371/journal.pone.0286151 (PMC10198486; doi:10.1371/journal.pone.0286151)
Supplement: S5 Table — (DOCX) [file pone.0286151.s005.docx]

**S5 Table.** Model selection results for linear mixed models of incubation bout duration for (**A**) Mallard, (**B**) Gadwall, and (**C**) Cinnamon teal in Suisun Marsh, California, 2015-2019. All models include nest identification as a random effect to account for non-independence of repeated measures within nests. Models in the table include the top-ranked models representing 0.99 cumulative model weights, the null model (random effect and error only), and in bold, the top model and models that are the same as the top model but with one of the variables removed. Nest Stage is a categorical variable that includes the egg-laying stage and the incubation stage after the clutch has been completed. Final Clutch Size is the clutch size after all eggs have been laid. NID = Nest initiation date. Ambient Temp is the temperature around the nest at the time the incubation bout began. cosHour and sinHour are the cosine and sine of the hour in radians at the time the incubation bout began. Variables with a superscript of 2 or 3 indicate quadratic and cubic trends, respectively.

| Model | k | -2LogL | AIC_c_ | ΔAIC_c_ | *w_i_* | Evidence ratio |
| --- | --- | --- | --- | --- | --- | --- |
| (**A**) Mallard |  |  |  |  |  |  |
| **Nest Stage + Final Clutch Size + Nest Age + Ambient Temp + Ambient Temp×Ambient Temp + sinHour + cosHour + Final Clutch Size×Nest Stage + Nest Stage×Nest Age** | **12** | **39074.93** | **39098.96** | **0.00** | **0.32** | **1.00** |
| Nest Stage + Final Clutch Size + Nest Age + NID + Ambient Temp + Ambient Temp×Ambient Temp + sinHour + cosHour + Final Clutch Size×Nest Stage + Nest Stage×Nest Age | 13 | 39074.12 | 39100.15 | 1.19 | 0.17 | 1.81 |
| Nest Stage + Final Clutch Size + Nest Age + NID + Ambient Temp + Ambient Temp×Ambient Temp + sinHour + cosHour + Final Clutch Size×Nest Stage + Final Clutch Size×NID + Nest Stage×Nest Age | 14 | 39073.10 | 39101.13 | 2.17 | 0.11 | 2.96 |
| Nest Stage + Final Clutch Size + Nest Age + Ambient Temp + sinHour + cosHour + Final Clutch Size×Nest Stage + Nest Stage×Nest Age | 11 | 39079.17 | 39101.19 | 2.23 | 0.10 | 3.05 |
| Nest Stage + Final Clutch Size + Nest Age + NID + NID×NID + Ambient Temp + Ambient Temp×Ambient Temp + sinHour + cosHour + Final Clutch Size×Nest Stage + Nest Stage×Nest Age | 14 | 39073.24 | 39101.28 | 2.32 | 0.10 | 3.18 |
| Nest Stage + Final Clutch Size + Nest Age + NID + Ambient Temp + sinHour + cosHour + Final Clutch Size×Nest Stage + Nest Stage×Nest Age | 12 | 39077.64 | 39101.67 | 2.71 | 0.08 | 3.87 |
| Nest Stage + Final Clutch Size + Nest Age + NID + Ambient Temp + sinHour + cosHour + Final Clutch Size×Nest Stage + Final Clutch Size×NID + Nest Stage×Nest Age | 13 | 39076.62 | 39102.65 | 3.69 | 0.05 | 6.34 |
| Nest Stage + Final Clutch Size + Nest Age + NID + NID×NID + Ambient Temp + sinHour + cosHour + Final Clutch Size×Nest Stage + Nest Stage×Nest Age | 13 | 39076.80 | 39102.83 | 3.87 | 0.05 | 6.93 |
| Nest Stage + Final Clutch Size + Nest Age + NID + NID×NID + Ambient Temp + Ambient Temp×Ambient Temp + sinHour + cosHour + Final Clutch Size×Nest Stage + Final Clutch Size×NID + Final Clutch Size×NID×NID + Nest Stage×Nest Age | 16 | 39072.79 | 39104.84 | 5.88 | 0.02 | 18.91 |
| **Nest Stage + Final Clutch Size + Nest Age + Ambient Temp + Ambient Temp×Ambient Temp + sinHour + cosHour + Nest Stage×Nest Age** | **11** | **39104.57** | **39126.59** | **27.63** | **0.00** | **1.00×10^6^** |
| **Nest Stage + Nest Age + Ambient Temp + Ambient Temp×Ambient Temp + sinHour + cosHour + Nest Stage×Nest Age** | **10** | **39118.90** | **39138.92** | **39.96** | **0.00** | **4.75×10^8^** |
| **Nest Stage + Final Clutch Size + Nest Age + sinHour + cosHour + Final Clutch Size×Nest Stage + Nest Stage×Nest Age** | **10** | **39119.40** | **39139.42** | **40.46** | **0.00** | **6.09×10^8^** |
| **Nest Stage + Final Clutch Size + Nest Age + Ambient Temp + Ambient Temp×Ambient Temp + sinHour + cosHour + Final Clutch Size×Nest Stage** | **11** | **39193.19** | **39215.22** | **116.26** | **0.00** | **1.76×10^25^** |
| **Nest Stage + Final Clutch Size + Ambient Temp + Ambient Temp×Ambient Temp + sinHour + cosHour + Final Clutch Size×Nest Stage** | **10** | **39200.42** | **39220.44** | **121.47** | **0.00** | **2.39×10^26^** |
| **Final Clutch Size + Nest Age + Ambient Temp + Ambient Temp×Ambient Temp + sinHour + cosHour** | **9** | **39246.23** | **39264.25** | **165.28** | **0.00** | **7.78×10^35^** |
| **Nest Stage + Final Clutch Size + Nest Age + Ambient Temp + Ambient Temp×Ambient Temp + Final Clutch Size×Nest Stage + Nest Stage×Nest Age** | **10** | **39576.79** | **39596.81** | **497.85** | **0.00** | **1.28×10^108^** |
| Null | 3 | 40024.27 | 40030.28 | 931.31 | 0.00 | 1.71×10^202^ |
| (**B**) Gadwall |  |  |  |  |  |  |
| **Nest Stage + Final Clutch Size + Nest Age + Ambient Temp + Ambient Temp×Ambient Temp + sinHour + cosHour + Final Clutch Size×Nest Stage + Nest Stage×Nest Age** | **12** | **25364.41** | **25388.46** | **0.00** | **0.48** | **1.00** |
| Nest Stage + Final Clutch Size + Nest Age + NID + Ambient Temp + Ambient Temp×Ambient Temp + sinHour + cosHour + Final Clutch Size×Nest Stage + Nest Stage×Nest Age | 13 | 25363.90 | 25389.95 | 1.50 | 0.23 | 2.11 |
| Nest Stage + Final Clutch Size + Nest Age + NID + NID×NID + Ambient Temp + Ambient Temp×Ambient Temp + sinHour + cosHour + Final Clutch Size×Nest Stage + Nest Stage×Nest Age | 14 | 25363.24 | 25391.30 | 2.84 | 0.12 | 4.13 |
| Nest Stage + Final Clutch Size + Nest Age + NID + Ambient Temp + Ambient Temp×Ambient Temp + sinHour + cosHour + Final Clutch Size×Nest Stage + Final Clutch Size×NID + Nest Stage×Nest Age | 14 | 25363.78 | 25391.84 | 3.38 | 0.09 | 5.43 |
| **Nest Stage + Final Clutch Size + Nest Age + Ambient Temp + Ambient Temp×Ambient Temp + sinHour + cosHour + Nest Stage×Nest Age** | **11** | **25372.10** | **25394.13** | **5.67** | **0.03** | **17.07** |
| Nest Stage + Final Clutch Size + Nest Age + NID + NID×NID + Ambient Temp + Ambient Temp×Ambient Temp + sinHour + cosHour + Final Clutch Size×Nest Stage + Final Clutch Size×NID + Final Clutch Size×NID×NID + Nest Stage×Nest Age | 16 | 25362.64 | 25394.72 | 6.26 | 0.02 | 22.88 |
| Nest Stage + Final Clutch Size + Nest Age + NID + Ambient Temp + Ambient Temp×Ambient Temp + sinHour + cosHour + Nest Stage×Nest Age | 12 | 25371.52 | 25395.56 | 7.11 | 0.01 | 34.93 |
| Nest Stage + Final Clutch Size + Nest Age + NID + NID×NID + Ambient Temp + Ambient Temp×Ambient Temp + sinHour + cosHour + Nest Stage×Nest Age | 13 | 25370.99 | 25397.04 | 8.58 | 0.01 | 72.93 |
| Nest Stage + Final Clutch Size + Nest Age + NID + Ambient Temp + Ambient Temp×Ambient Temp + sinHour + cosHour + Final Clutch Size×NID + Nest Stage×Nest Age | 13 | 25371.43 | 25397.48 | 9.03 | 0.01 | 91.17 |
| **Nest Stage + Nest Age + Ambient Temp + Ambient Temp×Ambient Temp + sinHour + cosHour + Nest Stage×Nest Age** | **10** | **25383.84** | **25403.87** | **15.42** | **0.00** | **2.23×10^3^** |
| **Nest Stage + Final Clutch Size + Nest Age + Ambient Temp + Ambient Temp×Ambient Temp + sinHour + cosHour + Final Clutch Size×Nest Stage** | **11** | **25387.74** | **25409.78** | **21.32** | **0.00** | **4.26×10^4^** |
| **Nest Stage + Final Clutch Size + Ambient Temp + Ambient Temp×Ambient Temp + sinHour + cosHour + Final Clutch Size×Nest Stage** | **10** | **25397.04** | **25417.07** | **28.61** | **0.00** | **1.63×10^6^** |
| **Nest Stage + Final Clutch Size + Nest Age + sinHour + cosHour + Final Clutch Size×Nest Stage + Nest Stage×Nest Age** | **10** | **25410.38** | **25430.42** | **41.95758** | **0.00** | **1.29×10^9^** |
| **Final Clutch Size + Nest Age + Ambient Temp + Ambient Temp×Ambient Temp + sinHour + cosHour** | **9** | **25439.65** | **25457.68** | **69.22138** | 0.00 | **1.07×10^15^** |
| **Nest Stage + Final Clutch Size + Nest Age + Ambient Temp + Ambient Temp×Ambient Temp + Final Clutch Size×Nest Stage + Nest Stage×Nest Age** | **10** | **26519.41** | **26539.44** | **1150.984** | **0.00** | **8.6×10^249^** |
| Null | 3 | 27019.8 | 27025.81 | 1637.348 | 0.00 | >3.56×10^307^ |
| (**C**) Cinnamon teal |  |  |  |  |  |  |
| **Stage + Nest Age + Nest Stage×Nest Age** | 6 | 4020.61 | 4032.68 | 0.00 | 0.99 | 1.00 |
| **Stage + Nest Age** | 5 | 4034.42 | 4044.46 | 11.78 | 0.00 | 362.29 |
| Stage + Nest Age^3^ | 7 | 4030.73 | 4044.82 | 12.14 | 0.00 | 432.16 |
| Stage + Nest Age^2^ | 6 | 4034.42 | 4046.48 | 13.80 | 0.00 | 994.07 |
| **Stage** | 4 | 4039.83 | 4047.86 | 15.18 | 0.00 | 1982.51 |
| Nest Age^2^ | 5 | 4041.29 | 4051.34 | 18.66 | 0.00 | 11258.22 |
| Null | 3 | 4045.52 | 4051.53 | 18.85 | 0.00 | 12425.10 |
| **Nest Age** | 4 | 4044.02 | 4052.05 | 19.37 | 0.00 | 16082.73 |
| Nest Age^3^ | 6 | 4041.20 | 4053.27 | 20.59 | 0.00 | 29553.96 |

^a^ The number of parameters in the model.

^b^ Akaike’s Information Criterion corrected for small sample size.

^c^ The difference in the AIC_c_ values of the current model and the model with the lowest AIC_c_.

^d^ Akaike model weight. The likelihood of the model given the data, relative to other models in the candidate set.

^e^ The weight of evidence that the model with the lowest AIC_c_ value is better than the current model.
